# A proposal of a simplified grading and echo-based staging of aortic valve stenosis to streamline management

**DOI:** 10.1186/s44156-024-00064-x

**Published:** 2024-11-04

**Authors:** Attila Kardos, Mani A. Vannan

**Affiliations:** 1 Department of Cardiology, Translational Cardiovascular Research Group, Milton Keynes University Hospital NHS Foundation Trust, Milton Keynes, UK; 2 Faculty of Medicine and Health Sciences, University of Buckingham, Buckingham, UK; 3 Marcus Heart Valve Center, Piedmont Heart Institute, Atlanta, GA USA

**Keywords:** Grading, Staging, Aortic valve stenosis, Continuity equation, Simplification

## Abstract

In this paper we discuss the relevance of continuity equation based aortic valve area (AVA) calculation as a robust parameter suitable for accurate grading of aortic stenosis (AS) irrespective of flow conditions. Combining the AVA-based *grading* and echocardiography-based *staging*, can provide with the most comprehensive clinical assessment of patients with AS and preserved left ventricular systolic function to streamline management decisions.

**Supplementary Information:**

The online version contains supplementary material available at 10.1186/s44156-024-00064-x.

## Introduction

Aortic valve stenosis (AS) is characterised by describing the structure of the aortic valve (AV) i.e. its cuspidality, texture (thickness, fibro-calcific changes), the AV area (AVA) and the haemodynamic features (flow velocity and pressure gradient) [1, 2]. Due to its calcification associated with calcific AS planimetric assessment of the anatomic orifice area is very challenging and correlation with severity and prognosis is unclear [3, 4]. The effective orifice area (EOA) assessed by the ultrasound-based continuity equation utilising the principles of the fluid dynamic is the closest to that to the invasive area measurement by the Gorlin formula [4, 5].

In this paper we submit that echocardiographically calculated AVA as a measure of severity of AS combined with echocardiography-based *staging*, which accounts for the extra valvular consequences of AS provides with the most comprehensive clinical assessment of patients with AS and preserved left ventricular systolic function to streamline management decisions.

### The proposal

**Fig. 1:**
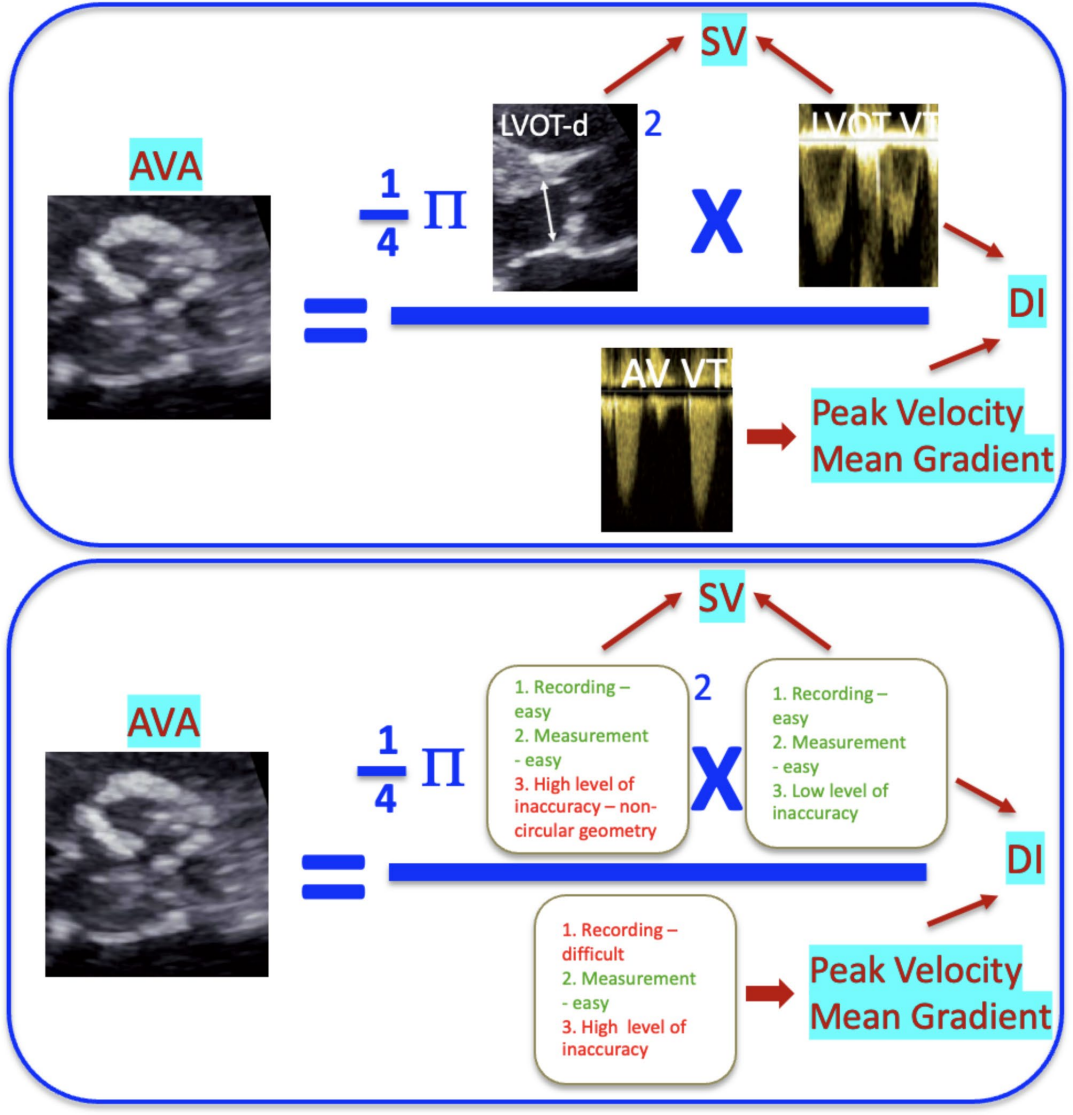
Simplified Grading of Aortic Stenosis. AVA by the Continuity Equation – a composite value for AS grading AVA: aortic valve area, AS: aortic stenosis, SV: stroke volume, DI: dimensionless index (AVAVTI/LVOTVTI), LVOT-VTI: left ventricular outflow tract velocity time integral, AVVTI: Transvalvular velocity time integral

Calculation of the AVA by the continuity equation has three distinct components (Fig. 1): left ventricular (LV) outflow tract (LVOT) area derived from the LVOT diameter from the parasternal long axis view, and two Doppler velocity measurements one of which in the LVOT (LVOT-VTI) and the other across the aortic valve (AV-VTI). Based on the fluid dynamics and the mass preservation law the AVA = LVOTA x LVOT-VTI /AV-VTI or LV-SV/AV-VTI. This equation incorporates several haemodynamic features of the aortic valve; the LV stroke volume, the dimensionless index, peak velocity, and mean gradient (Fig. 1). The continuity equation-based AVA includes all the three flow components and makes it attractive to be the sole parameter to be used for grading the severity of the valve lesion. This, of course, assumes that all the component elements are accurately measured, and errors are minimised (Supplement Figs. 1–4) [2]. 

Indeed, professional societies after the validation of the continuity equation by the Gorlin formula in 1988 by Oh et al. [5] proposed its sole use for grading aortic stenosis and the grade of severe AS was defined as AVA ≤ 1.0 cm^2^ in the American Heart Association/American College of Cardiology valvular guidelines in 1998 [6]. Definition of mild (> 1.5cm^2^), moderate (1.1-1.5cm^2^), severe AS(≤ 1cm^2^) without the inclusion of the peak velocity or mean gradient for grading aortic stenosis was recommended until the review and the updated guidelines in 2006 [7] based on limited observational outcome data [8].

**Fig. 2:**
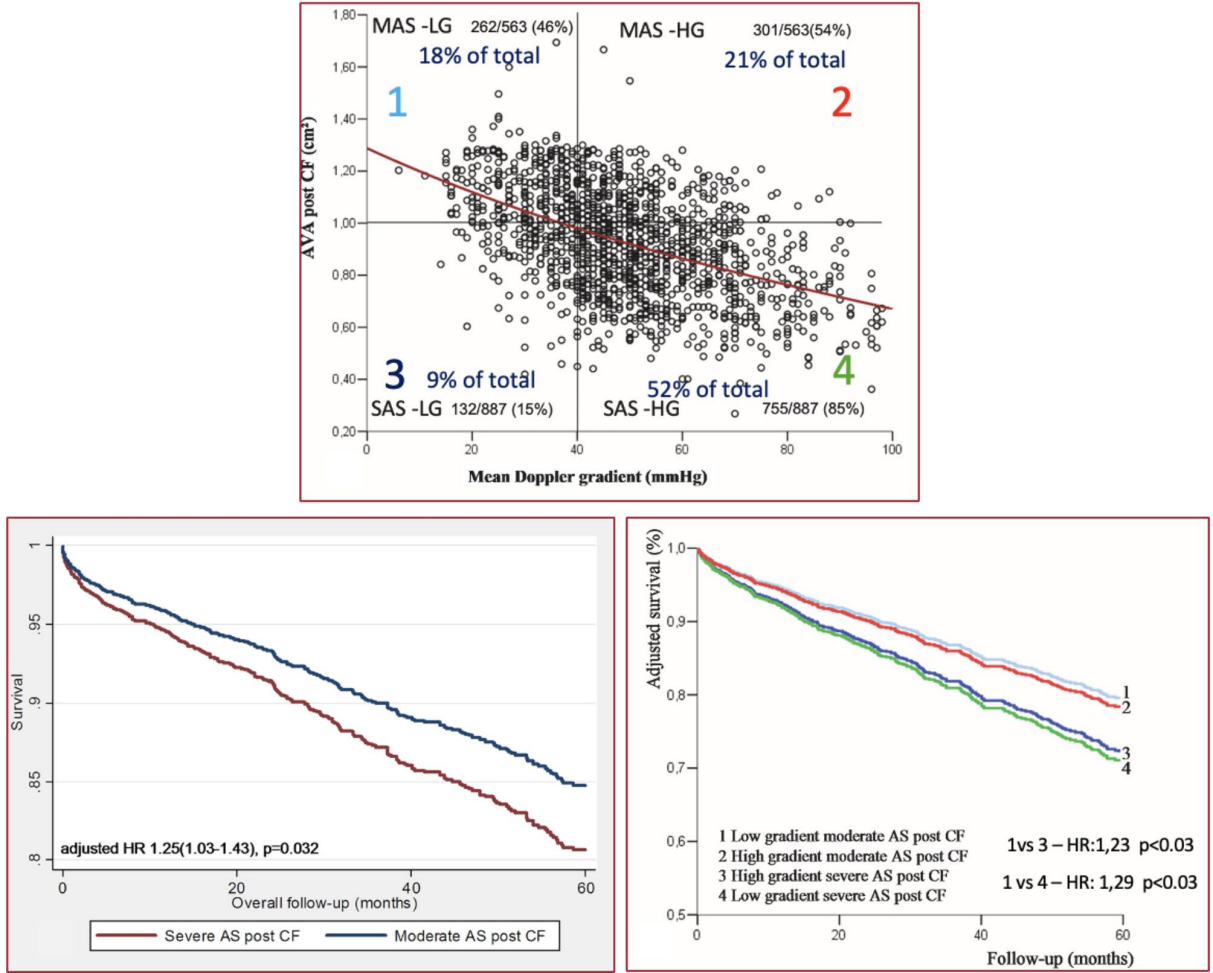
AVA - the predicting power of outcome after reclassification by the Correction Factor AVA: aortic valve area, SAS: severe aortic stenosis, MAS: moderate aortic stenosis, HG: high gradient (≥ 40mmHg), LG: low gradient (< 40mmHg) HR: hazard ratio, CF: correction factor (reference: 13)

The seminal paper in 2007 has led to definition of low flow, low gradient phenotype despite of the EOA of < 1cm^2^, highlighting the load dependency of those parameters [9]. It also allowed to further study the value of the flow (stroke volume index, SVi) on outcome in paradoxical low flow – low gradient severe AS patients with normal left ventricular systolic function. The AVA remained a powerful parameter in grading and predicting all-cause mortality [9]. Inconsistencies in grading aortic stenosis was reported in 2008 highlighting the discrepancies of the cut off points of peak velocity ≥ 4 m/s and the mean pressure gradient of ≥ 40mmHg associated with the AVA < 1cm^2^ as marker of severe AS. Using the Gorlin formula Minners et al. showed that severe AS based on AVA ≤ 1cm^2^ was the most prevalent parameter (69% of cases) compared with the peak velocity ≥ 4cm^2^ (45%) and mean pressure gradient ≥ 40 mmHg (40%) only, predominantly due their flow dependency [10]. This was further supported by our observation in 1450 severe AS patients with preserved left ventricular systolic function after applying the validated correction factor (CF) of 1.13 for overcoming the inaccuracies of LVOT area measurements stemming from the non-circular geometry of this structure. The reclassified moderate AS cohort (39% of the total) showed better 5 years all-cause mortality compared to severe AS based on AVA [11, 12]. More importantly we found that the mean gradient (defined as ≥ 40mmHg or < 40mmHg) did not discriminate between all-cause mortality but only AVA did (Fig. 2). Patients with severe AS irrespective of the mean gradient (high or low) had the same outcome HR: 0.97[0.68–1.40]; *p* = 0.88). The reclassified moderate AS (post CF) had better outcome than severe AS (post CF) independent from the mean pressure gradient (Fig. 2). Further evidence showed that SVi was a prognostic indicator in AS [12, 13] which is one of the components of the continuity equation to calculate AVA.

In addition to the simplified *grading* of AS by the continuity equation, we would propose the implementation of the echocardiography-based *staging* in the prognostication and management decision of AS by addressing the extra-valvular cardiac damage. Staging had been recently proposed based on prior AV intervention of RCTs and subsequently big registry data and showed a good discrimination of 1- and 5-year mortality with more advanced stages. These stages were developed based on the following echocardiographic parameters: LV-mass, LV diastolic function: grade 2 or higher, LV ejection fraction < 60%, or global LV longitudinal strain ≥-15%, left atrial volume index ≥ 35ml/m^2^, mitral regurgitation ≥ moderate, systolic pulmonary hypertension ≥ 60mmHg, tricuspid regurgitation ≥ moderate, right ventricular systolic impairment: Tricuspid Annular Plane Systolic Excursion < 17 mm, tricuspid annulus e’<9.5 cm/s, right ventricular SVi:<30 ml/m2 [13–17]. Although the retrospective analysis of the staging proposal is very favorable prospective validation is essential prior its recommendation for clinical implementation.

**Fig. 3:**
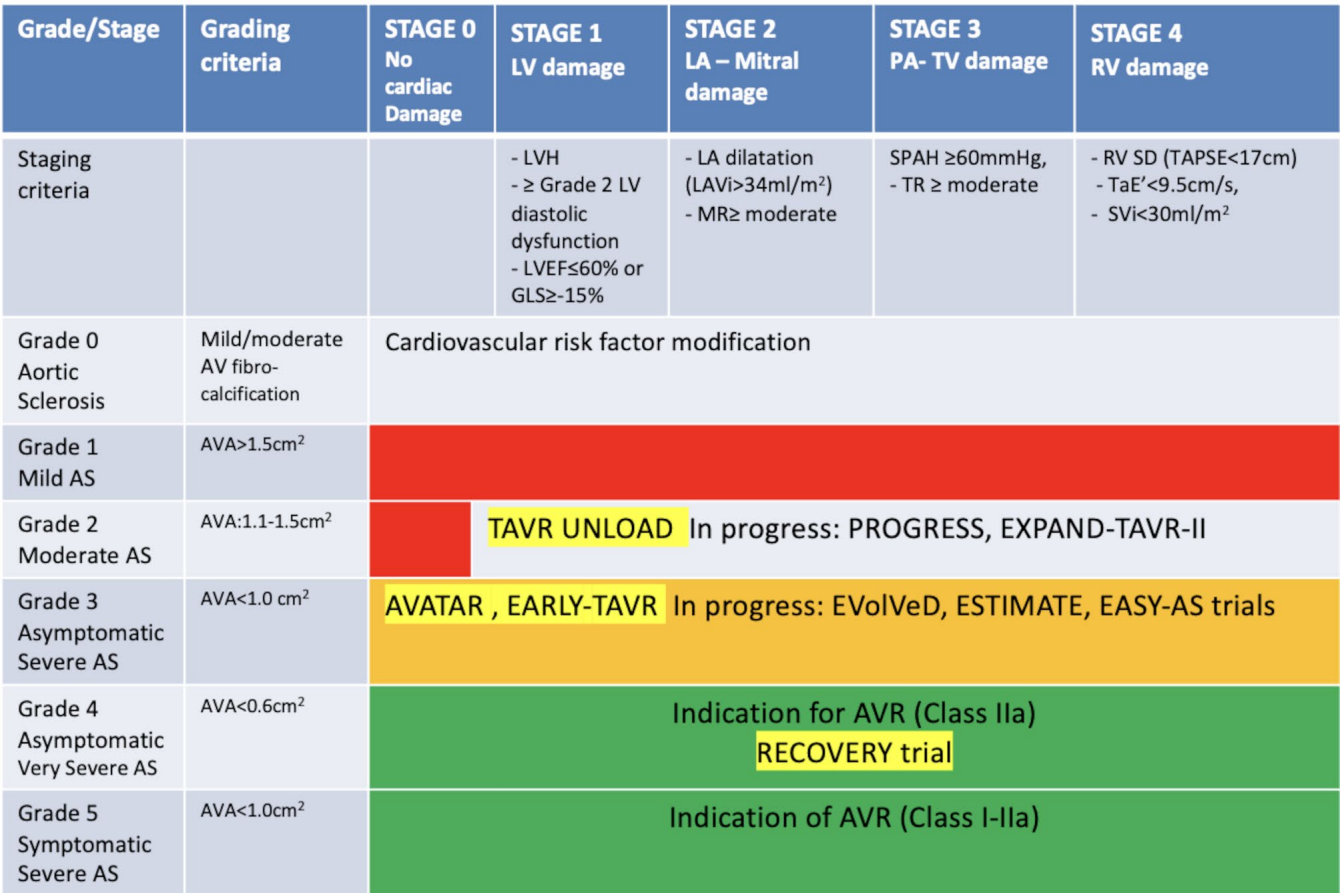
Proposal - Grading and Staging aortic valve stenosis in patients with preserved left ventricular ejection fraction PROGRESS- NCT04889872 at Clinical Trials.gov, TAVR UNLOAD: NCT02661451, EXPAND-TAVR-II: NCT05149755, AVATAR: NCT02436655, EARLY-TAVR: NCT03042104, EVolVeD: NCT03094143, ESTIMATE: NCT02627391, EASY-AS: NCT04204915, RECOVERY: NCT01161732 AVR: aortic valve replacement AVA: aortic valve area, LVH: left ventricular hypertrophy, LVEF: left ventricular ejection fraction, GLS: LV global longitudinal strain, LA: left atrium, LAVi: left atrial volume index, MR: mitral regurgitation, SPAH: systolic pulmonary pressure, TR: tricuspid regurgitation, RV: right ventricle, SD: systolic dysfunction, TaE’: Tricuspid annular E’ Doppler velocity, SVi: Stroke volume index, AS: aortic stenosis

We therefore propose a simplified disease grading using the continuity equation-based AVA (as a multiparametric echocardiographic measurement) to classify the severity of aortic stenosis and the echocardiography-based staging to describe extra-valvular sequalae to assist in the selection and the timing of AV intervention (Fig. 3). Ongoing RCT in different grade and stage of AS phenotypes are listed in Fig. 3.

### Learning points

1. Continuity equation-based AVA (effective orifice area) provides with the most comprehensive assessment of the AV – encompassing several haemodynamic and anatomical parameters.
2. Grading of AS should be based on effective orifice area (AVA by continuity equation with careful attention to acquisition and measurements of the parameters, as prescribed in the guidelines).
3. Simplified grading by AVA and echocardiography-based staging may streamline selection and management of patients with AS with preserved LV ejection fraction.
4. The diagnostic workup of patients with severe AS with low flow and low gradient due to reduced LV ejection fraction should follow the current European and American Valvular heart disease guidelines.

## Electronic supplementary material

Below is the link to the electronic supplementary material.

Suppl. Figure 1. How to record and measure LVOT-diameter?. LVOT: left ventricular outflow tract

Suppl. Figure 2. How to record and measure LVOT-VTI?. LVOT: left ventricular outflow tract, VTI: velocity time integral. PW: pulse wave, SV: stroke volume, VTI: velocity time integral

Suppl. Figure 3. How to record and measure AV-VTI?. AV: transvalvular, VTI: velocity time integral, RPS: right parasternal space. CW: continuous wave

Suppl. Figure 4. How to assess aortic valve anatomy?

## Data Availability

No datasets were generated or analysed during the current study.
