# Supplementary figures and images for: A proposal of a simplified grading and echo-based staging of aortic valve stenosis to streamline management
Source: Echo Res Pract. 2024 Nov 4;11:29. doi: 10.1186/s44156-024-00064-x (PMC11533394; doi:10.1186/s44156-024-00064-x)

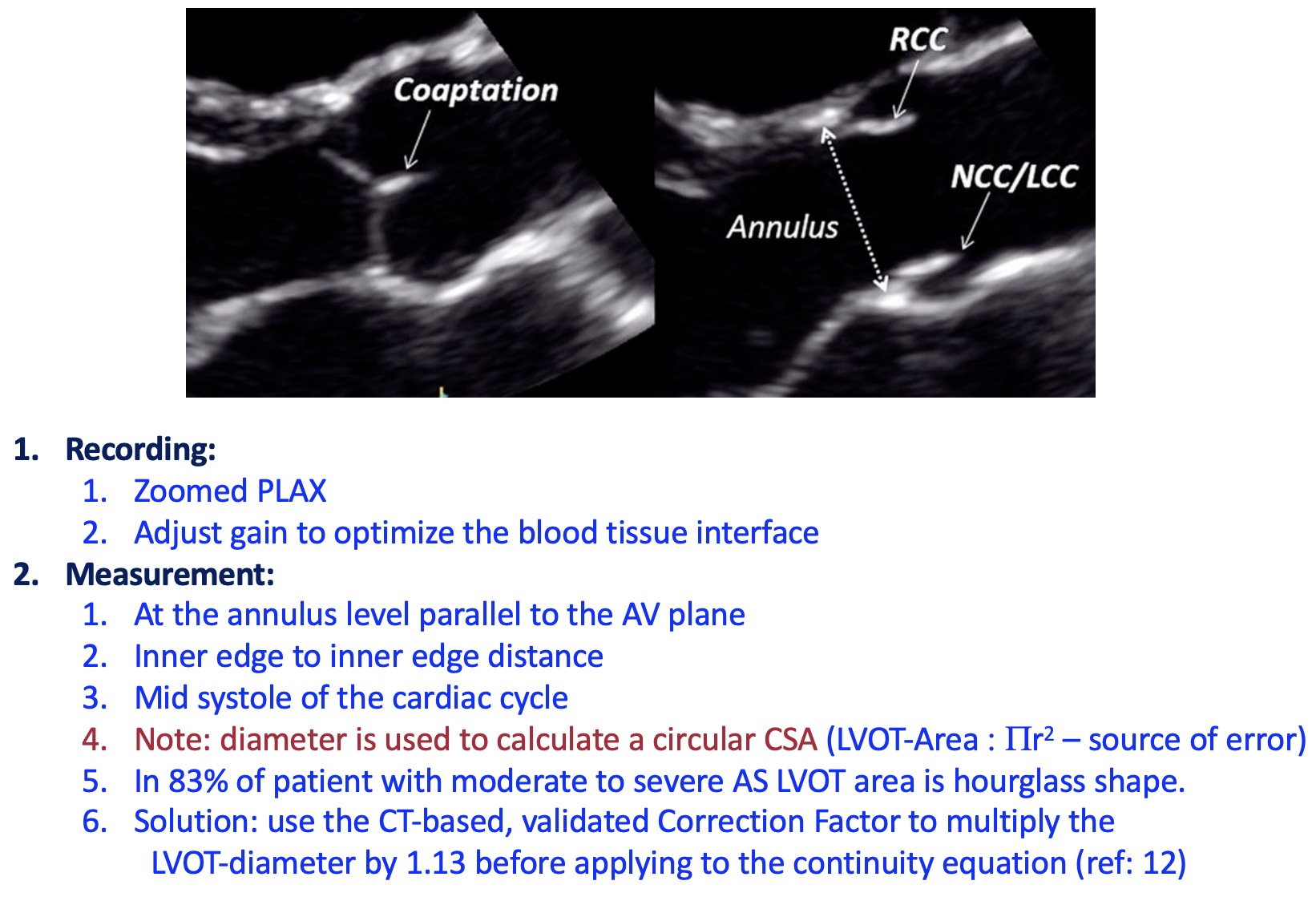

Supplement: Supplementary file 1 — Suppl. Figure 1. How to record and measure LVOT-diameter?. LVOT: left ventricular outflow tract [file 44156_2024_64_MOESM1_ESM.jpg]

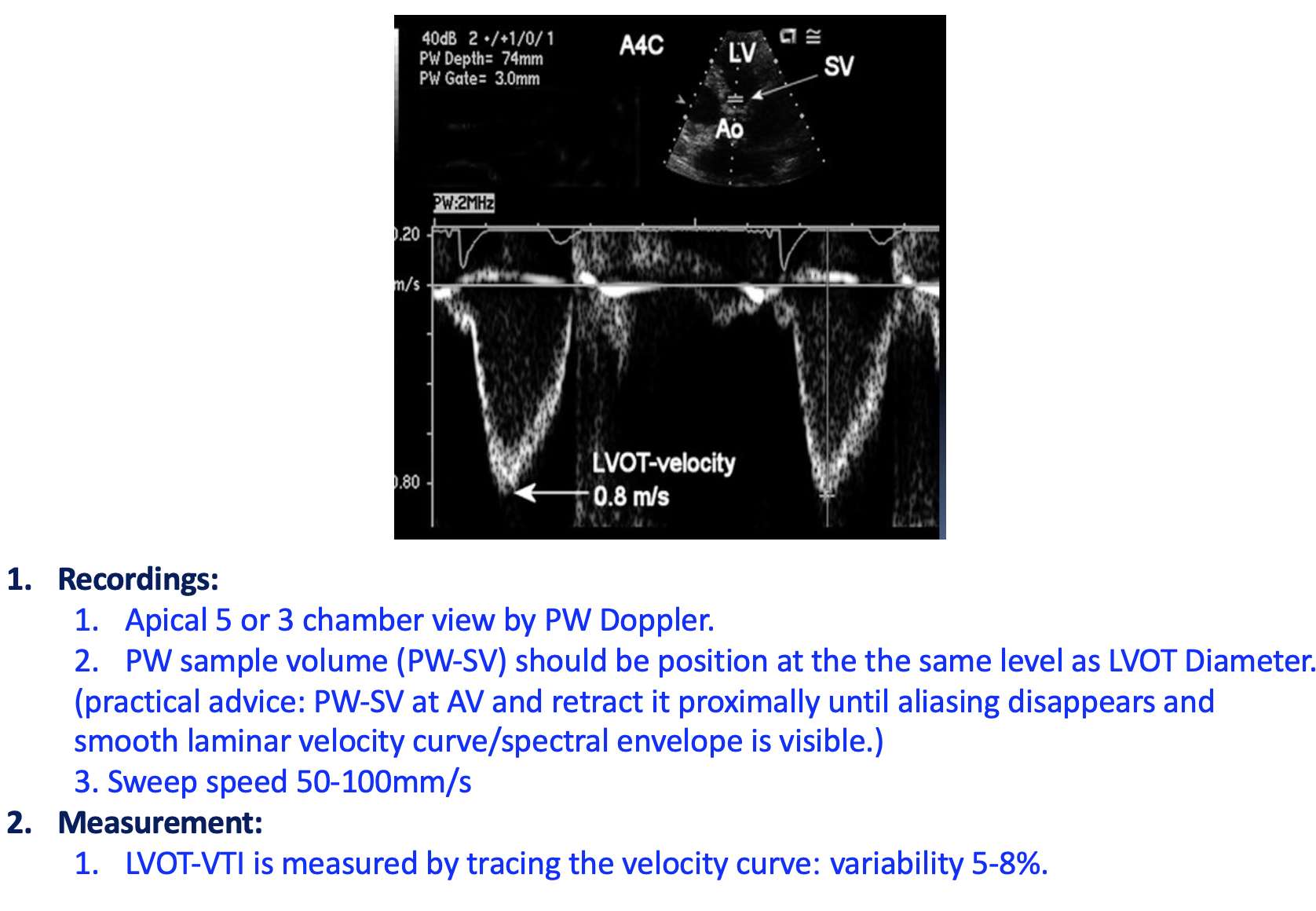

Supplement: Supplementary file 2 — Suppl. Figure 2. How to record and measure LVOT-VTI?. LVOT: left ventricular outflow tract, VTI: velocity time integral. PW: pulse wave, SV: stroke volume, VTI: velocity time integral [file 44156_2024_64_MOESM2_ESM.jpg]

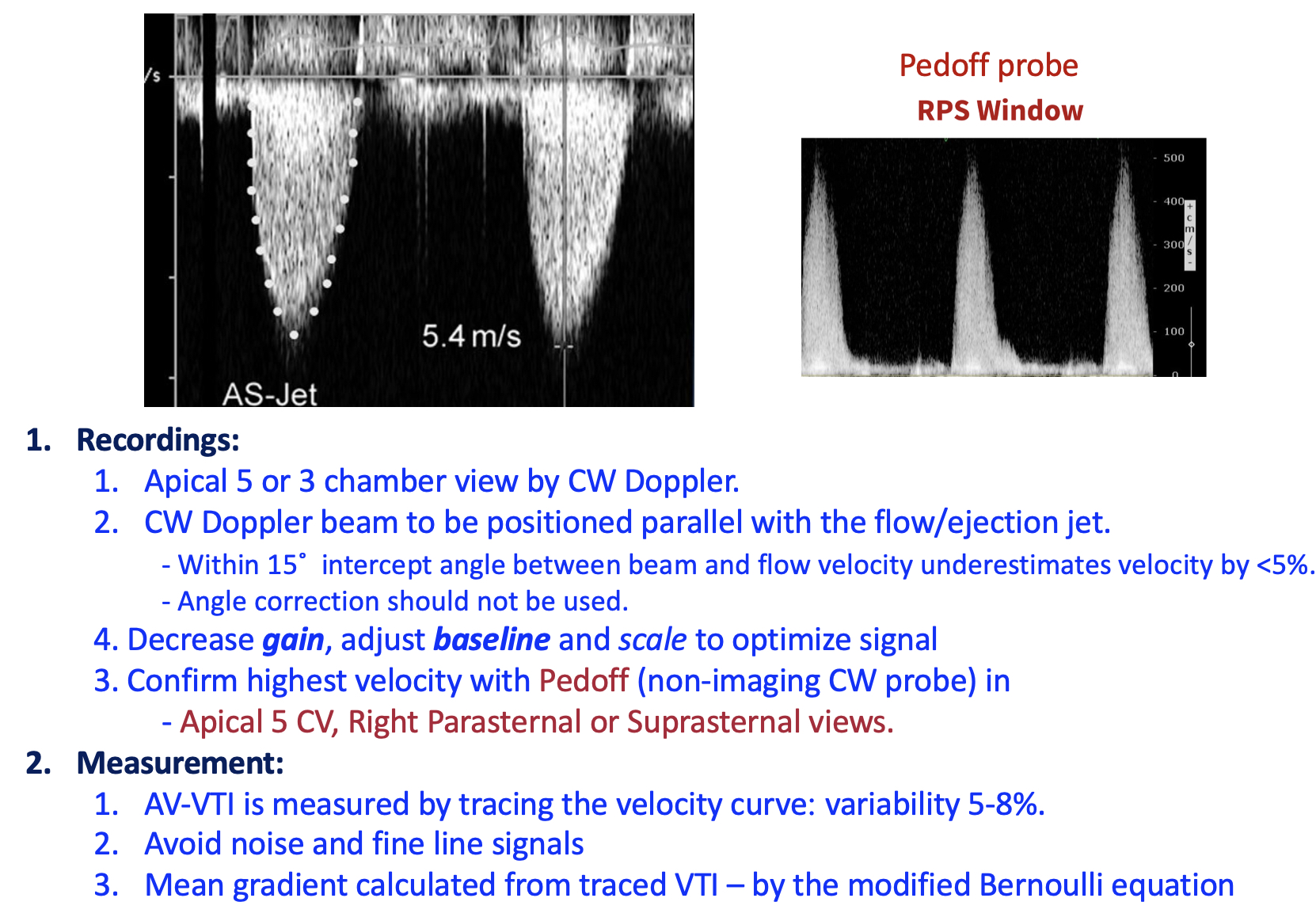

Supplement: Supplementary file 3 — Suppl. Figure 3. How to record and measure AV-VTI?. AV: transvalvular, VTI: velocity time integral, RPS: right parasternal space. CW: continuous wave [file 44156_2024_64_MOESM3_ESM.jpg]

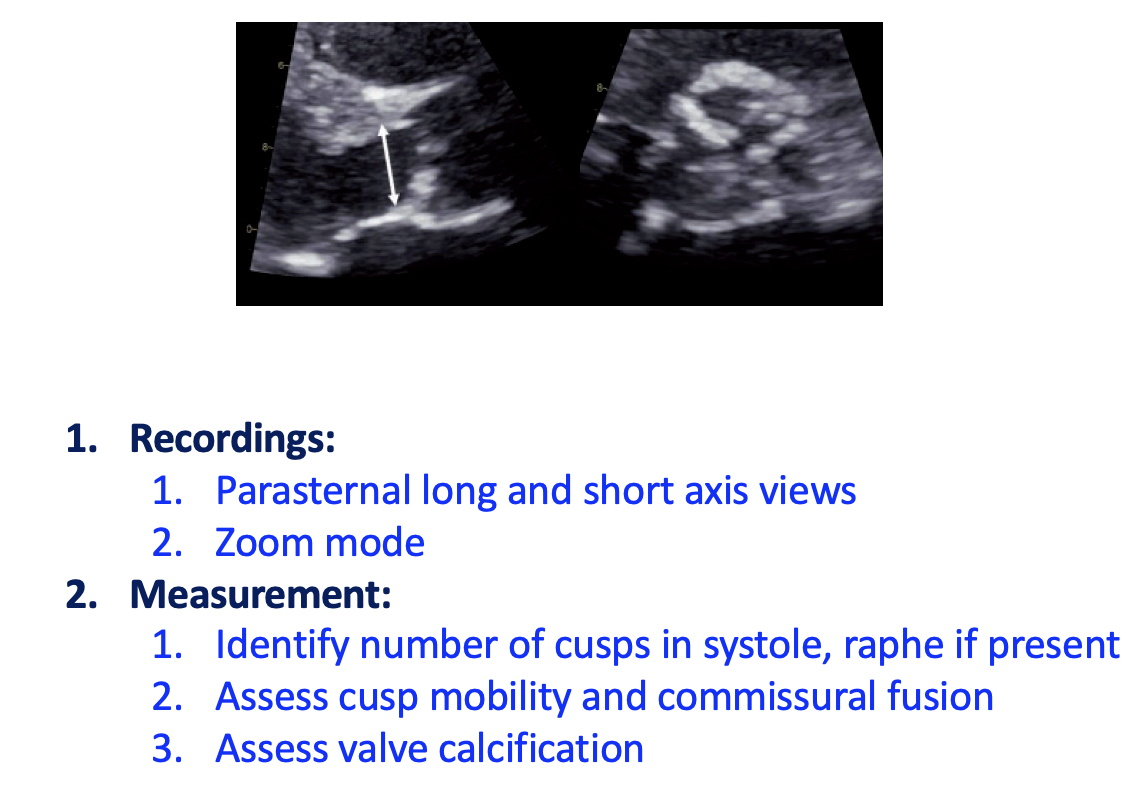

Supplement: Supplementary file 4 — Suppl. Figure 4. How to assess aortic valve anatomy? [file 44156_2024_64_MOESM4_ESM.jpg]
